# Is your dog empathic? Developing a Dog Emotional Reactivity Survey

**DOI:** 10.1371/journal.pone.0170397

**Published:** 2017-02-13

**Authors:** Flóra Szánthó, Ádám Miklósi, Enikő Kubinyi

**Affiliations:** 1 Eötvös Loránd University, Department of Ethology, Pázmány P. s. 1/c, Budapest, Hungary; 2 MTA–ELTE Comparative Ethological Research Group, Pázmány P. s. 1/c, Budapest, Hungary; Hungarian Academy of Sciences, HUNGARY

## Abstract

Dogs' seemingly empathic behaviour attracts general and scientific attention alike. Behaviour tests are usually not sufficiently realistic to evoke empathic-like behaviour; therefore we decided to ask owners about their experiences with their dogs in emotionally loaded situations. Owners from Hungary (N = 591) and from Germany (N = 2283) were asked to rate their level of agreement on a 1–5 Likert scale with statements about the reactivity of their dogs to their emotions and to other dogs’ behaviour. We created two scales with satisfactory internal reliability: reactivity to the owner’s emotion and reactivity to other dogs’ behaviour. Based on an owner-dog personality matching theory, we hypothesised that the owner’s empathy, as measured by the subscale on the cooperativeness character factor of the human personality, will correlate with their dog’s emotional reactivity in emotionally loaded situations. In addition we also examined how anthropomorphism, contagious yawning, attitude toward the dog are related to emotional reactivity in dogs as perceived by the owner. In addition we examined how owners rate dog pictures. We found that the scale scores were largely independent from demographic and environmental variables like breed, sex, age, age at acquiring, keeping practices, training experiences and owner's age. However, anthropomorphic and emotional attitude of the owners probably biased the responses. In the German sample more empathic owners reported to have more emotionally reactive dog, as expected by the personality matching theory. More empathic owners reported to have fewer problems with their dogs and they rated a puppy picture as more cute in both countries. 62% of owners from Hungary and 36% of owner from Germany agreed with the statement “My dog is more important for me than any human being”. In Germany, more empathic owners agreed less with this statement and indicated that their dogs have a tendency for contagious yawning. Owners whose attitudes toward their dogs were anthropomorphic (agreed more with the statement that “My dog thinks like a child”), perceived their dogs as more reactive to their emotions. This findings highlights the importance of testing the attitudes of the respondents when they assess the personality and the emotions of animals. The criterion validity of the Dog Emotional Reactivity Survey should be confirmed by objective behavioural tests.

## Introduction

The dogs' ancestor was originally a social species and during domestication they successfully integrated into human society. Dogs show special sociocognitive abilities (for example they are able to follow human communicative cues like pointing [1], gazing [2], they look at the owner when they cannot reach a required object [3]), and they display some "infantile" morphological features [4]. The human-dog relationship shares many common features with the parent-child relationship [5]. There are similarities in the attachment behaviour, and the relationship is asymmetrical and dependence-based in the case of both infants and dogs [6].

Some studies have raised the possibility that dogs may empathize with humans. Sümegi [7] examined the presence of emotional contagion (an elementary mechanism of empathy [8]) between dogs and owners, and tested whether dogs show some signs of taking on their owner’s current affective state. The results suggested that the owner’s state of anxiety was contagious to their dog and the emotional contagion could be tracked by measuring changes in the dog’s memory performance [7].

Several studies tested dogs’ reactions to human crying. Yong and Ruffman [9] used three auditory stimuli: a human infant crying, a human infant babbling, and computer-generated “white noise”. They measured not only behavioural responses, but also cortisol level changes in both humans and dogs. They found that only crying elicited an increase in cortisol levels in dogs and humans, as well as a combination of behaviour in dogs involving submissiveness and not surprisingly, alertness. These findings suggest that dogs can recognise and react to human internal state changes with an increased stress response. The authors tried to exclude the possibility that the dogs’ behaviour was the result of earlier experience, such as being reinforced by humans comforting them. They argued that dogs living without children would have limited opportunities for being reinforced in such situations. However, findings showed that the dogs with no experience with human children still had a cortisol and behavioural response to crying. It is, however, also possible that the vocalisation of the human infant is acoustically similar to some species specific vocalisation in dogs (e.g. whining). Thus, dogs may respond on the basis of specific vocal features and do not ‘recognise’ human baby crying as such.

In another test adults pretended to cry in the presence of the dog [10]. Dogs oriented more (looked at, approached, touched) and behaved submissively (licked, nuzzled) toward the owner or an experimenter when they pretended to cry, compared to when they were humming or talking. These results indicated that the crying carried greater emotional valence for the dogs. However, it is possible that dogs may have previously received positive reinforcement for approaching crying individuals. A pet dog who approaches a distressed human family member is likely to be positively reinforced by receiving affection [10]. However, it is possible that dogs’ pattern of response was driven by comfort-seeking or distress caused by the simulated situation [11].

In contrast to dog studies, in human studies the conventional method to measure reactions to others’ emotions is the use of questionnaires. Cloninger et al. [12] described empathy as a feeling of unity or identification with other people, that allows improved communication and compassion for others. A frequently used survey for measuring empathy in humans, the Empathy Quotient (EQ), was developed by Baron-Cohen and Wheelwright [13].

Surprisingly, similar questionnaire has not been applied in dogs, although surveys have proved to be appropriate means for investigating broad traits such as personality traits in dogs in several studies (reviewed in Kubinyi et al. [14]). Owners have the opportunity to observe their dogs in several contexts for an extended time, and the fact that owners’ responses correlated with the opinion of another observer, indicate that they are able to reliably evaluate their dog’s behaviour [15].

In our study we aim to assess how owners describe their dog’s behaviour in some emotionally loaded situations. We hypothesised that the owner’s empathy, as measured by the subscale on the cooperativeness character factor of the human personality [12], correlates with their dog’s emotional reactivity in emotionally loaded situations. The basis of this hypothesis is the owner-dog personality matching theory: by the means of questionnaires, Turcsán et al. [16] showed that the owner-dog partnership resembles the human-human partnership. Dog owners perceive similarity in their own and their dogs’ personality, similarly to the way that humans perceive similarities between themselves and their partners. The authors argued that living with a partner who has similar personality traits may reduce the risk of conflicts, because it is easier to predict the partner’s future behaviours. It is also possible that conflicts may be reduced if one perceives his partner as similar.

We also examine several interesting phenomena which may be related to empathy and/or which may bias the scores of the respondents about the emotional reactivity of dogs.

1 Hills found that high levels of empathy are positively associated with some degree of belief in the mental experience of animals [17] (anthropomorphism). Therefore we asked owners how they assess the mental abilities of their dog.
2 Studies have suggested that there is a link between contagious yawning and the level of empathy in humans [8] [18], [19]. The absence of contagious yawning in humans with autism spectrum disorder implies that it may be related to the capacity for empathy [18]. While spontaneous yawning is widespread among vertebrate species [20], contagious yawning has been reported to occur only in some non-human species: chimpanzees (*Pan troglodytes*) [21], [22], bonobos (*Pan paniscus*) [23] and gelada baboons (*Theropithecus gelada*), when they observe a conspecific yawning [24].

Some studies indicate that dogs also show contagious yawning, but there is contradictory evidence, with others finding no evidence, or that results are strongly dependent on different factors (such as familiarity, and the presence of visual and/or auditory cues). The first study investigating contagious yawning in dogs from Joly-Mascheroni et al. [25] showed that a high proportion of the subjects (72%) yawned after observing a human experimenter acting out a yawn (with visual and auditory cues). In the study of Silva et al. [26] the results were similar using only auditory cues. Romero et al. found that dogs yawned more frequently when watching a familiar model than an unfamiliar one, demonstrating that the contagiousness of yawning in dogs correlated with the level of emotional proximity [27]. On the contrary, Harr et al. failed to replicate Joly-Mascheroni et al.’s finding, however they used only visual cues [28]. O’Hara and Reeve [29] also did not find a contagious yawning effect in dogs, and they rejected the hypothesis of empathy-based yawning contagion. In their study, familiarity did not induce more yawning than unfamiliarity; indeed, the trend was in the opposite direction. In this study we aim to investigate whether owners perceive a link between their dog's emotional reactivity and contagious yawning.

3 In a recent study, Lehmann et al. [30] investigated the relationship between empathy and the baby schema effect (BSE: infantile appearance features, which evoke protecting and nurturing mechanisms) [31]. The respondents had to rate photographs on a scale from 1 (not at all), to 5 (very much), on how they were emotionally affected by seven adult animals (cat, dog, horse, chicken, lion, elephant and rabbit), and a corresponding juvenile version of these animals. The ratings of juvenile animals’ photographs were significantly higher than that of the adult pictures. According to the results, humans were not only sensitive to the human, but also to the animal BSE. Most importantly, gender and empathy consistently and strongly correlated with BSE. Women rated all pictures more positively than men, moreover women scored higher on the Basic Empathy Scale [30].
4 Attitude toward the dog and environmental variables might also affect the assessment of the dog's emotional reactivity. We ask owners how important the dog is in their life and whether they have a child, among other factors.
5 Another aspect of this study was collecting the same kind of data in two countries (Germany and Hungary) to examine whether in spite of the cultural differences in pet-related practices and attitudes we find similar correlations between the scales [32]. Most studies on dog keeping practices and attitudes focus on a single cultural group or country, while cross-cultural studies of dog-keeping practices are limited ([33]). In a previous study Turcsán et al. [16] showed cultural differences in the personality matching between owners and dogs in two different countries (Austria and Hungary). Possible cultural differences in factors like dog keeping practices, dogs’ general role, shared activities, or factors affecting the dog choice may explain the differences in the similarity pattern to the owner. Moreover cultural differences might also explain why the behaviour of pets themselves differs across cultures [32]. Wan et al. [32] asked owners of the German shepherd dog in Hungary and the USA about their dog- keeping practices, as well as their dogs’ behavioural attributes. They found that certain characteristics of the dog–owner relationship differed between Hungary and the USA. German shepherd owners from the USA were more likely than owners from Hungary to offer terms like ‘‘pet,” ‘‘companion,” or ‘‘family member” as the reason for having their dogs, to keep their German shepherd dogs indoors and to train their dogs for more varieties of activities. In addition they rated their dogs as more confident and aggressive compared to German shepherd dog owners from Hungary.

## Methods

### Subjects

Hungarian owners of 591 dogs (six owners with two dogs, and one with three dogs) filled in an online questionnaire, which was advertised on the website of the Family Dog Project, Budapest, Hungary and on an online social networking service. The questionnaire was available from 06 June 2014 to 5 August 2014. 17 owners filled out the questionnaire twice (for the same dog), with more than half a year between the two entries. Questionnaires with missing data were included in the final results and also in cross-country comparisons to increase sample size (sample sizes are indicated with the results).

We translated the Hungarian questionnaire to German. The German questionnaire was available from 15 April 2014 to 7 January 2015, and 2285 replies were collected.

Participants provided written consent to their participation. Our Consent Form was based on the Ethical Codex of the Hungarian Psychologists (2004). We took special care to ensure that the consent process was understood completely by the participant. In the Consent Form participants were informed about the identity of the researchers, the aim, procedure, location, expected time commitment of the experiment, the handling of personal and research data, and data reuse. The information included the participant's right to withdraw their consent at any time. Participants could easily (and without penalty) decline to participate and could ask not to use or delete data collected during the experiments. In online surveys, participants indicated their approval by answering ‘yes’ for the question whether they had read and approved the Consent Form.

The filling out of the questionnaires was anonymous so the study did not violate respondents' privacy. We confirm that the procedures comply with national and EU legislation.

### Procedure

The Dog Emotional Reactivity Survey consisted of several parts: General Questions, Attitude toward Dogs; Owner Empathy Scale; Dog’s Reactivity to the Owner’s Emotion; Dog’s Reactivity to other Dog’s Behaviour. The items are listed in Table 1.

**Table 1:** Scales and items of the Dog Emotional Reactivity Survey.

**General Questions**
1. Name of the dog
2. Breed of the dog (purebred; mixed-breed)
3. Sex and neuter status of the dog (intact male; intact female; neutered male; neutered female)
4. Keeping place of the dog (indoor, outdoor, both)
5. Training level of the dog (no formal training, basic training (obedience), specific training (agility))
6. Gender of the owner
7. Whether the owner Has a child (yes; no)
8.Age of the dog (in years)
9. Age at acquiring (number of months)
10. Age of the owner
**Attitude toward Dogs (1: I disagree strongly… 5: I agree strongly)**
1. I have no problems with my dog at all. (NO PROBLEM)
2. My dog thinks like a child. (THINK CHILD)
3. My dog means more to me than any human being. (VIP)
4. Due to my dog I feel that somebody loves me. (LIKE ME)
5. The puppy in the picture is cute. (PUPPY PIC)
6. The adult dog in the picture is cute. (ADULT DOG PIC)
7. My dog tends to yawn when I yawn. (YAWNING)
**Owner’s Empathy Scale (OE; 1: I disagree strongly… 5: I agree strongly)**
1. I consider others' feelings to be alike to mine.
2. I am not able to understand the majority of people.
3. People tell me their feelings easily.
4. Usually I try to imagine myself in the situation of the other if I want to understand him/her better.
5. I frequently try to put aside my own opinion in order to understand others' feelings.
6. I would prefer if people did not talk so much.
7. I think it is not possible to share our feelings with somebody who did not have the same experience.
8. I enjoy it a lot if I can take care of others.
9. I quickly realise if somebody in a group feels good or whether they are uncomfortable.
**Dog’s Reactivity to the Owner’s Emotion Scale (DROE; 1: I disagree strongly… 5: I agree strongly)**
1. My dog is frightened if I am afraid of something.
2. If I am surprised, my dog also seems to be surprised.
3. If I am happy, my dog comes to me, and may even seek body contact with me.
4. When I am frightened of something, my dog does not realize it.
5. If I am sad, my dog comes to me, and may even seek body contact with me.
6. My dog does not react to an unexpected event even if I am surprised.
7. My dog is calm even if I laugh loudly.
**Dog’ Reactivity to other Dog’s Behaviour Scale (DRDB; 1: I disagree strongly… 5: I agree strongly)**
1. My dog remains calm even if it sees that another dog avoids something (for example the administration of a medicine)
2. My dog shows no reaction when another dog is frightened.
3. If a dog is crying, or whining, my dog is also distracted, and may start to cry.
4. My dog is nervous when other dogs around him are excited, or aggressive.
5. My dog does not realize if something unexpected happens and other dogs are surprised.

One item of the Attitude toward Dogs part (‘My dog is more important for me than any human being’) was modified from Templer’s Pet Attitude Scale (original question: My pet means more to me than any of my friends.) [34]. We included in the Attitude toward Dogs part whether the dog is disposed to yawn when the owner yawns, based on the assumed link between contagious yawning and empathy [18].

The Owner Empathy (OE) Scale was developed based on the Cooperativeness character factor from the Temperament and Character Inventory by Cloninger et al. [12] in English, and we used a previously translated Hungarian version from Rózsa et al. [35]. The questions measure how much people are cooperative, socially tolerant, empathic, helpful and compassionate, but we chose only the empathy and social acceptance subscale for our scale (C1, C2). We added two items from the Empathy Quotient questionnaire (EQ), [13] to increase the number of human empathy questions to create two almost equally long questionnaire parts.

To develop the Dog’s Reactivity to the Owner’s Emotion Scale (DROE) and the Dog’s Reactivity to other Dogs’ Behaviour Scale (DRDB), we adapted a parent questionnaire developed by Rieffe et al. [36], who measured the empathy-related behaviours of young children. We chose this questionnaire because there is some evidence for an analogy between human-child and owner-dog bonding [6]. The EmQue consists of 20 items representing three facets of empathy that should be observable in young children: (a) Emotion Contagion, (b) Attention to Others’ Feelings, and (c) Prosocial Actions. From the questionnaires, we selected questions which targeted the six basic emotions (anger, disgust, fear, happiness, sadness and surprise) [37]), and choose only those questions, which were suitable to characterise the dog-owner relationship. Initially, a total of 12 items formed the Dog’s Reactivity to the Owner’s Emotion Scale and the Dog’s Reactivity to other Dogs’ Behaviour Scale. In a pilot study on 171 subjects, we ran a factor analysis, and used only the questions which had high loadings (>0.4) on the two factors. As a result the final DROE Scales consisted of seven items and the DRDB consisted of five items (Table 1).

**Fig 1:**
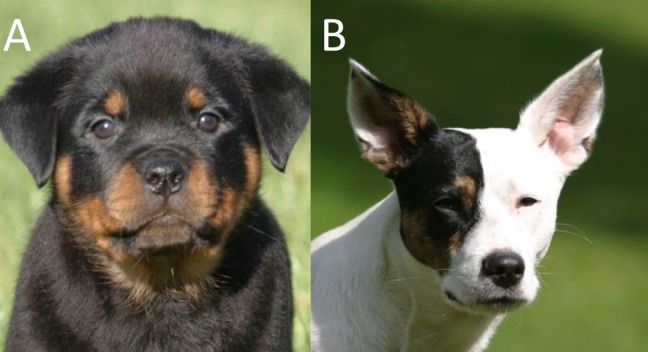
Dog pictures rated by dog’s owners. a) Rottweiler puppy, b) Adult mixed-breed dog. Permission to republish as CC BY granted by Debbie Cornell-Charneski and Jesko Wilke, original copyright holders of photos A and B, respectively.

Participants were also asked to rate pictures of a Rottweiler puppy and an adult mixed-breed dog (Fig 1). We assumed that owners who liked the dog puppy pictures more, had higher empathy scores and reported that their dog had a high tendency for emotional reactivity (based on Lehman et al. [30]; see above).

### Statistical analysis

SPSS 21 was used for the analysis. Chi^2^ tests with Z post hoc test (p < 0.05) and t-tests were used to compare the demographic variables of the samples between the two countries.

Cronbach’s alpha (CA) was calculated to assess the internal reliability of the three scales (OE, DROE, DRDB) separately for the two countries and the test-retest reliability on the Hungarian Sample.

We investigated the correlations between the three scales with Spearman correlation.

Univariate general linear models tested the main effects of the independent variables on the three scales (OE, DROE, and DRDB) for each country and scale separately. Dog age, age at acquisition, age of the owner, and the seven statements from the Attitude towards the Dog part of the questionnaire were covariates ((1) I have no problems with my dog at all; (2) My dog thinks like a child; (3) My dog means more to me than any human being; (4) Due to my dog I feel that somebody loves me; (5) The puppy in the picture is cute {picture of a Rottweiler puppy}; (6) The adult dog in the picture is cute {picture of an adult mixed breed}; (7) My dog tends to yawn when I yawn), and the sex of the dog, training experience, gender of the owner, and keeping conditions were fixed factors. Bonferroni correction was used to correct for multiple comparisons. Only *p* values lower than 0.003 were considered as significant according to the Bonferoni correction.

## Results

### Differences between the countries

German owners kept mixed-breeds and neutered males more often than Hungarian owners. Moreover, Germans kept dogs indoors more often, their dogs were more trained and more owners had at least one child. They were older, acquired their dogs at an earlier age, and their dogs were older than the dogs of the Hungarians. German owners agreed less with these statements: “I have no problems with my dog at all; My dog thinks like a child; My dog means more to me than any human being; The puppy on the picture is cute”. German owners scored themselves as less empathic than Hungarians, and scored theirs dogs lower on the DROE and DRDB Scales. However the effect sizes were small or negligible (Table 2).

**Table 2:** Descriptive statistics of the subjects and comparison of the items and scores between Hungary and Germany. Only p < 0.003 were considered as significant (highlighted in bold). Effect sizes considered at least as 'small' are highlighted in bold.

		Hungary		Germany				
		N	%	N	%	P	chi2	
Breed	Mixed-breed	174	29.4%	918	40.2%	**<0.001**	23.1	
	Purebred	417	70.6%	1365	59.8%			
Sex and neuter status	Intact male	152	25.8%	546	23.9%	**<0.001**	23.7	
	Intact female	137	23.2%	467	20.5%			
	Neutered male	105	17.8%	627	27.5%			
	Neutered female	196	33.2%	643	28.2%			
Keeping conditions	Indoor	282	47.7%	1510	66.9%	**<0.001**	73.7	
	Outdoor too/only	309	52.3%	748	33.1%			
Training	No formal training	243	41.3%	658	28.8%	**<0.001**	33.6	
	Basic	177	30.1%	831	36.4%			
	Special	169	28.7%	794	34.8%			
Owner gender	Man	64	10.9%	183	8.0%	0.03	4.8	
	Woman	525	89.1%	2100	92.0%			
Children	No	315	70.9%	1348	59.0%	**<0.001 **	22.1	
	Yes	129	29.1%	935	41.0%			
		N	Mean (SD)	N	Mean (SD)	P	F	Eta Squared
Age of the dog (years)		589	4.63 (3.22)	2282	5.56 (3.42)	**<0.001**	35.38	**0.012**
Age at acquiring (in months)		590	7.61 (14.95)	2283	5.82 (11.34)	**<0.001**	10.12	0.004
Age of the owner (years)		588	35.3 (11.95)	2276	41.88 (11.11)	**<0.001**	158.94	**0.053**
NO PROBLEM		591	3.87 (1.01)	2283	3.69 (1.05)	**<0.001**	14.14	0.005
THINK CHILD		589	3.46 (1.31)	2283	2.71 (1.26)	**<0.001**	164.08	**0.054**
VIP		591	3.59 (1.15)	2283	3.02 (1.18)	**<0.001**	113.07	**0.038**
LIKE ME		591	3.27 (1.32)	2283	3.08 (1.30)	<0.01	9.45	0.003
PUPPY PIC		589	4.21 (1.00)	2283	3.95 (1.05)	**<0.001**	28.89	**0.010**
ADULT DOG PIC		444	3.61 (1.21)	2283	3.40 (1.16)	**<0.001**	12.46	0.005
YAWNING		590	2.75 (1.34)	2283	2.85 (1.31)	0.106	2.61	0.001
OE Scale		591	3.69 (0.58)	2283	3.46 (0.50)	**<0.001**	88.72	**0.030**
DROE Scale		589	3.77 (0.71)	2283	3.63 (0.74)	**<0.001**	18.41	0.006
DRDB Scale		591	3.53 (0.83)	2283	3.37 (0.79)	**<0.001**	17.80	0.006

Abbreviations: NO PROBLEM: “I have no problems with my dog at all.”, THINK CHILD: “My dog thinks like a child.”, VIP: “My dog means more to me than any human being“, LIKE ME: “Due to my dog I feel that somebody loves me”, PUPPY PIC: The puppy in the picture is cute, ADULT DOG PIC: The adult dog in the picture is cute, YAWNING:” My dog tends to yawn when I yawn”, OE Scale: Owner’s Empathy Scale, DROE Scale: Dog’s Reactions to the Owner’s Emotions Scale, DRDB Scale: Dog’ Reactions to Other Dog’s Behaviour Scale.

### Internal consistency of the scales

In the Hungarian sample, Cronbach's alpha values were 0.67 for the Owner’s Empathy Scale, 0.74 for the Dog’s Reactivity to the Owner’s Emotion Scale and 0.64 for the Dog’s Reactivity to Dogs’ Behaviour Scale. In the German sample, CA values were 0.58 for the Owner Empathy Scale, 0.73 for the Dog’s Reactivity to the Owner’s Emotion Scale, and 0.63 for the Dog’s Reactivity to Dogs’ Behaviour Scale. Although CA values below 0.7 indicate that more related items should be added, previous studies have shown that lower values are generally also acceptable (e.g. [38]).

### Test-retest reliability

The test–retest reliability in the Hungarian Sample (N = 17) for the Owner Empathy Scale was high (CA = 0.847), but lower for the Dog’s Reactivity to the Owner’s Emotion Scale (CA = 0.591), and for the Dog’s Reactivity to Dogs’ Behaviour Scale (CA = 0.644).

### Correlations between the scales

All scales including the Owner Empathy Scale (OE), the Dog’s Reactivity to the Owner’s Emotion Scale (DROE), the Dog’s Reactivity to other Dogs’ Behaviour Scale (DRDB), were correlated with each other, in both the Hungarian and German samples. However, three of the correlations were below the minimum threshold of *r* = 0.2, indicating that there was no real relationship between the OE scale and DROE/DRDB in the Hungarian sample, and the OE and the DRDB in the German sample. However, there was a weak correlation between OE and DROE in the German sample, such that, owners who rated themselves as empathic perceived their dog as more reactive to their emotions. Dogs were similarly reactive to the owners’ and to other dogs’ emotions/behaviours, according to the questionnaires filled out by the owners in both the German and Hungarian samples (*r* = <0.4) (Table 3).

**Table 3:** Correlations between the three scales of the survey. Correlation is significant at the 0.01 (**) or 0.05 (*) level. Correlations above the minimum threshold (r = 0.2) are highlighted in bold. Sample sizes are in brackets.

	Hungary (N = 591)		Germany (N = 2283)	
	DROE	DRDB	DROE	DRDB
OE	0.188**	0.090*	0.229**	0.093**
DROE		0.436**		0.478**

Abbreviations: OE Scale: Owner’s Empathy Scale, DROE Scale: Dog’s Reactions to the Owner’s Emotions Scale, DRDB Scale: Dog’ Reactions to Other Dog’s Behaviour Scale.

### Effects of independent variables on scales

#### Owner Empathy scale (OE)

More empathic owners had fewer problems with their dogs and they rated the puppy picture as more cute in both countries (Table 4).

**Table 4:** Relationship between the three Scales and the survey’s independent variables investigated with GLM. The direction of the relationship is indicated with + or–in brackets. Only differences with p < 0.003 were considered significant and are highlighted.

			Owner’s Empathy Scale (OE)						Dog’s Reactivity to the Owner’s Emotions Scale (DROE)						Dog’s Reactivity to Other Dogs’ Behaviour Scale (DRDB)						
			HUNGARIAN			GERMAN			HUNGARIAN			GERMAN			HUNGARIAN				GERMAN		
Variables	Df HU	Df G	F	Sig.	Partial Eta Squared	F	Sig.	Partial Eta Squared	F	Sig.	Partial Eta Squared	F	Sig.	Partial Eta Squared	F	Sig.		Partial Eta Squared	F	Sig.	Partial Eta Squared
BREED	1	1	.005	.943	.000	1.758	.185	.001	5.601	.018	.013	.392	.531	.000	7.154	.008	.017		3.791	.052	.002
SEX	3	3	.382	.766	.003	.069	.976	.000	.861	.461	.006	1.091	.352	.001	.625	.599	.004		.633	.594	.001
AGE	1	1	.104	.748	.000	.553	.457	.000	.031	.860	.000	6.915	.009	.003	.056	.814	.000		6.317	.012	.003
AGE AT ACQ	1	1	.642	.423	.002	.195	.659	.000	.720	.397	.002	.114	.736	.000	.273	.602	.001		.309	.578	.000
KEEPING	1	1	.008	.927	.000	1.810	.179	.001	.649	.421	.002	.115	.735	.000	1.164	.281	.003		.149	.700	.000
TRAINING	2	2	.831	.436	.004	4.167	.016	.004	.727	.484	.003	.707	.493	.001	1.080	.340	.005		.285	.752	.000
OWNER GENDER	1	1	2.556	.111	.006	6.310	.012	.003	1.402	.237	.003	9.881 (women +)	.002	.004	1.409	.236	.003		9.176 (women +)	.002	**.004**
OWNER AGE	1	1	.179	.672	.000	1.108	.293	.000	.043	.836	.000	1.737	.188	.001	1.776	.183	.004		1.321	.251	.001
CHILDREN	1	1	2.535	.112	.006	.666	.415	.000	.066	.797	.000	1.344	.246	.001	.003	.953	.000		2.471	.116	.001
NO PROBLEM	1	1	9.409 (-)	.002	**.022**	20.852 (-)	<0.001	.009	2.843	.092	.007	.004	.951	.000	8.188	.004	.019		81.578 (+)	<0.001	**.035**
THINK CHILD	1	1	1.986	.159	.005	5.133	.024	.002	17.761 (+)	<0.001	**.040**	42.889 (+)	<0.001	**.019**	7.602	.006	.018		19.247 (+)	<0.001	.009
VIP	1	1	6.598	.011	.015	34.717 (-)	<0.001	**.015**	1.337	.248	.003	.013	.909	.000	.117	.733	.000		1.600	.206	.001
LIKE ME	1	1	2.945	.087	.007	.483	.487	.000	6.527	.011	.015	56.213 (+)	<0.001	**.025**	.050	.823	.000		7.656	.006	.003
PUPPY PIC	1	1	13.277 (+)	<0.001	**.030**	39.033 (+)	<0.001	**.017**	.079	.779	.000	3.419	.065	.002	1.675	.196	.004		.104	.747	.000
ADULT DOG PIC	1	1	.602	.438	.001	2.783	.095	.001	1.113	.292	.003	4.530	.033	.002	.040	.842	.000		2.660	.103	.001
YAWNING	1	1	.018	.894	.000	38.141 (+)	<0.001	**.017**	18.577 (+)	<0.001	**.042**	183.084 (+)	<0.001	**.076**	3.618	.058	.008		98.580 (+)	<0.001	**.042**
Error	423	2230																			

Abbreviations: NO PROBLEM: “I have no problems with my dog at all.”, THINK CHILD: “My dog thinks like a child.”, VIP: “My dog means more to me than any human being“, LIKE ME: “Due to my dog I feel that somebody loves me”, PUPPY PIC: "The puppy in the picture is cute", ADULT DOG PIC: "The adult dog in the picture is cute", YAWNING:” My dog tends to yawn when I yawn”, OE Scale: Owner’s Empathy Scale, DROE Scale: Dog’s Reactions to the Owner’s Emotions Scale, DRDB Scale: Dog’ Reactions to Other Dog’s Behaviour Scale.

In Germany, in addition, more empathic owners agreed less with the statement “My dog is more important for me than any human being” and indicated that their dog tends to yawn when they yawn (Table 4).

#### Dog’s Reactivity to the Owner’s Emotions (DROE)

Owners who agreed more with the statement that “My dog thinks like a child” and/or indicated that their dog tends to yawn when they yawn scored their dog higher on the DROE Scale in both countries (Table 4).

In Germany, in addition, women scored their dogs higher on the DROE Scale than men. Owners who agreed more with the statements that “Due to my dog I feel that someone loves me” scored their dog higher on the DROE Scale (Table 4).

#### Dog’s Reactivity to other Dogs’ Behaviour (DRDB)

In the Hungarian sample we did not find links between the scale and the independent variables.

In Germany, women scored their dogs higher on the DRDB Scale than men. Owners who agreed more with the statements: “I have no problems with my dog”, “My dog thinks like a child”, and “My dog tends to yawn when I yawn” scored their dogs higher on the DRDB Scale (Table 4).

## Discussion

In this study we developed a survey to assess how owners perceive their dogs’ reactions to human and conspecific emotional behaviour. Previous studies have used behavioural observations by manipulating test situations to provoke behavioural reactions from the dogs. Although the dogs’ reactions may have several possible explanations which might clarify their behaviour, without the need for emotional contagion or empathy, results regarding dogs’ abilities to empathise with humans have been inconclusive. However, if asked, dog owners tend to believe that their dogs can empathize with them. Our study is the first owner questionnaire survey, which aimed at measuring dog’s emotional reactivity.

The correlation between the dog’s two scales (DROE and DRDB) indicates that according to the owners, if a dog is reactive to humans’ emotions it is also reactive to other dogs’ behaviour.

Based on the personality matching hypothesis [16] we assumed that more empathic owners would indicate that their dogs are more reactive to their owner’s emotions or to other dog’s behaviour. In accordance with this assumption we found that there was a weak positive correlation between the empathy scale of German owners and the Dog’s Reactivity to the Owner’s Emotion Scale (DROE). The German sample allows a more robust interpretation of the results due to the fact that the sample size was higher than in the Hungarian sample.

According to a simple explanation owners may have a tendency to select dogs that possess traits which are similar to their own, either at the individual or at the breed level [16]. However, it is also a plausible explanation that the perception of empathic owners is biased, and they perceive their dogs as more reactive to emotions and behaviour. This is underlined by the finding that owners who agreed more with the statement that “My dog thinks like a child”, indicated that their dogs are more reactive to the owners’ emotions in both countries. Therefore, owners who tend to anthropomorphise their dogs also perceive their dog as more reactive to their emotions.

Although the anthropomorphising tendency and emotional bias of the owners may indicate that their perception of dog behaviour is biased, according to previous studies owner’s evaluation about their dogs’ behaviour was found to be generally reliable [16]. In Turcsán et al.’s study [16], not only the owners filled out the questionnaire about their dog’s behaviour, but also a member from the same household. The authors found positive associations between peer and self-ratings, and owners and dogs were assessed as being similar also by independent peer raters (in harmony with the results of [39]). However, there might be a general human tendency to perceive owners and dogs as similar.

The survey was filled out in two countries, and although the Hungarian and the German dog owner populations differed in almost all demographic measures (age, gender proportions, frequency of having a child, etc.), the three scales of the questionnaire (measuring the owner’s empathy, the dog’s reactions to the owner’s emotions, and to other dogs’ behaviour), had similar internal consistencies in both samples, and the scales had similar relationship with the independent variables.

### Owner Empathy scale

Although this was not in the focus of the analysis, our data collection enabled us to compare our results about human empathy to the literature. From previous studies we know that some factors such as having a child, gender, and age positively correlate with human empathy levels ([40], [17]). For example, Paul (2000) [41] showed in a questionnaire study that respondents who currently had a child or children living at home with them scored significantly higher on the human-oriented empathy scale, than those who did not. However, our study does not support this result. A plausible hypothesis is that the empathy level of dog owners (who volunteered to fill in a questionnaire) is different from a representative sample.

Many studies have demonstrated that women tend to have higher scores than man in measures of empathy ([39], [29], [42], [43]). We did not detect gender differences in the Owner Empathy scale in the Hungarian sample or the German sample. However, in the German sample women tended to indicate that their dogs are more reactive to the owner’s emotions and to other dogs’ behaviour more than men.

In contrast to Paul’s study [41] we did not find correlations between the owners’ empathy level and age in either sample. Again, please note that our sample was not representative as only dog owners were included, who were mostly middle-aged women.

However, we could detect a link between the Animal Baby Schema Effect ([30] see above) and empathy in both samples. Owners who found the dog puppy picture “cute” were more empathic. Interestingly, there was no relationship between the adult dog picture and empathy level.

Our knowledge about the mechanism behind contagious yawning and the capacity to empathize with others is limited [18]. There are contradictory results in studies investigating contagious yawning in dogs, which may be explained by variations in the methodology used (visual and auditory cues: [25], visual cues: [28], auditory cues: [26]). In the present study, German and Hungarian owners evaluated similarly their dog’s tendency for contagious yawning, although an additional 15 variables out of 19 differed between the two populations. Interestingly, German owners with a higher empathy score rated their dogs as more responsive to contagious yawning. Note also that a surprisingly high number of owners (one third of the sample) agreed with the statement that their dogs show contagious yawning.

However, it is questionable whether (1) more empathic owners’ dogs do indeed show more contagious yawning (related to their higher emotional reaction in some emotionally loaded situations), or (2) more empathic owners are better observers of their dogs and perceive their yawning more often, or (3) more empathic owners overestimate the frequency of contagious yawning in dogs.

It is also interesting that more empathic owners indicated fewer problems with their dogs. It is possible that more empathic owners understand more about the needs of dogs and perceive dog-specific behaviour as less problematic. An alternative explanation is that more empathic owners socialize and train their dogs in a more appropriate way. Further studies should identify the causes and effects in this case.

In the German sample, more empathic owners agreed less with the statement that their dogs were more important for them than any human being, and in the Hungarian sample we detected a tendency in the same direction. This indicates that for more empathic owners’, human acquaintances are more important to them than their dogs. However, they do not anthropomorphise their dogs less, as we found a tendency for a positive correlation between owner empathy and the “My dog thinks like a child” item (but only in the German sample). Notably, almost half of our sample (62% of owners from Hungary and 36% of owner from Germany) agreed with the statement “My dog means more to me than any human being”. This is surprising even in a sample that may not be representative, as people who volunteer to fill out a questionnaire about dogs’ emotional reactivity are more interested in dogs than the average person.

According to Harrison and Hall’s study [44], empathy evolved to promote social well-being among human beings, and due to the need to understand the intentions and emotions of conspecifics [45]. Anthropomorphism is a by-product of empathy. Harrison and Hall [44] found near-perfect relationships between perceptions of an animal’s ability to communicate with humans (using loosely and strictly defined constructs to attempt to cover non-linguistic and linguistic facets), and perceived empathy for that animal, based on a questionnaire study. We found that Hungarian owners anthropomorphised their dogs more than German owners (Hungarian owners agreed more with the statement “My dog thinks like a child”), and they scored higher on the empathy scale. The results provide additional evidence for a link between the owners’ perceived empathy of their dog, and the anthropomorphic attitudes of the owner.

### DROE and DRDB scales

In the German sample older dogs tended to be less reactive toward their owner’s emotions. This finding could be interesting regarding the cognitive aspects of aging, although again it is possible that this finding reflects the owners’ beliefs that older dogs are less reactive. In humans, older adults tend to adopt less the perspective of others to understand their thoughts and feelings (i.e. they have reduced cognitive empathy), but their sympathy or vicarious experience of others’ emotions does not differ with age [46].

German women rated their dogs as more reactive to their emotions and to other dogs’ behaviour. Although it is possible that dogs owned by women are more reactive than that of men’s, there is no objective behavioural data to support this result. Questionnaire-based studies found that the dogs of female owners are more trainable, more sociable, less bold [47], and less disobedient [48] than those of male owners. In a behavioural test study, owner gender had no significant effect on salivary cortisol values during four test situations [49], but in another study women had less sociable-active dogs than men [50]. However, in the latter case results could be explained by the fact that only 22 dogs were evaluated and subjective items (e.g. sociable-distant, active-inactive, and cheerful-not cheerful) were used for scoring the dogs. It would be important to compare the behaviour of dogs belonging to men and women, by using more objective behavioural variables to prove that the differences between dogs perceived by male and female owners are valid.

German owners who perceived fewer problems in their dogs scored their dogs higher on the DRDB Scale. It is interesting that more reactive dogs were rated as less problematic, although, the owners of these dogs were also more empathic, which could bias their assessments of their dogs.

The owners perception of their dogs’ behaviour is probably biased by their anthropomorphic attitude, as noted above. Owners who agreed more with the statement that “My dog thinks like a child” scored their dog higher on the DROE Scale in both countries, and higher on the DRDB scale in Germany.

German owners who highly appreciated the emotional support function of their dog (agreed more with the statement that “Due to my dog I feel that someone loves me”), also scored their dog more highly in the DROE Scale. It is not surprising that owners who feel that their dog loves them also perceive that their dog is more reactive to their emotions. This indicates that “feeling loved” is partly explained by attention and emotional support from the other’s part.

Dogs that were perceived as more responsive to their owners’ yawns were rated as more reactive towards their owner’s emotions (in both countries) and towards other dogs behaviour (in the German sample). These findings may strengthen the association between empathy and contagious yawning [25–29]. However, in the German sample more empathic owners perceived their dog as inclined for contagious yawning which may suggest that responses are biased by the owner's personality.

In conclusion, although based on the internal consistency, the test-retest reliability and the similar relationships between scale scores and other variables in two countries the Dog Emotional Reactivity Survey is a good start for evaluating dogs’ reactivity to human emotions and to other dogs’ behaviour, due to the strong effect of owners' anthropomorphic and emotional attitudes objective behavioural tests should confirm the criterion validity of the survey.
